# Tensile Properties of 21-6-9 Austenitic Stainless Steel Built Using Laser Powder-Bed Fusion

**DOI:** 10.3390/ma14154280

**Published:** 2021-07-31

**Authors:** Magnus Neikter, Emil Edin, Sebastian Proper, Phavan Bhaskar, Gopi Krishna Nekkalapudi, Oscar Linde, Thomas Hansson, Robert Pederson

**Affiliations:** 1 Division of Subtractive and Additive Manufacturing, University West, 46132 Trollhättan, Sweden; phavan.bhaskar@student.hv.se (P.B.); gopi-krishna.nekkalapudi@student.hv.se (G.K.N.); thomas.hansson@hv.se (T.H.); robert.pederson@hv.se (R.P.); 2 Division of Materials Science, Luleå University of Technology, 97187 Luleå, Sweden; emil.edin@ltu.se; 3 Research Institutes of Sweden, 41314 Gothenburg, Sweden; sebastian.proper@ri.se; 4 GKN Aerospace Sweden AB, 46181 Trollhättan, Sweden; oscar.linde@gknaerospace.com

**Keywords:** stainless steel, process parameters, laser powder-bed fusion (LPBF), alloy 21-6-9, design of experiment (DOE)

## Abstract

Alloy 21-6-9 is an austenitic stainless steel with high strength, thermal stability at high temperatures, and retained toughness at cryogenic temperatures. This type of steel has been used for aerospace applications for decades, using traditional manufacturing processes. However, limited research has been conducted on this alloy manufactured using laser powder-bed fusion (LPBF)**.** Therefore, in this work, a design of experiment (DOE) was performed to obtain optimized process parameters with regard to low porosity. Once the optimized parameters were established, horizontal and vertical blanks were built to investigate the mechanical properties and potential anisotropic behavior. As this alloy is exposed to elevated temperatures in industrial applications, the effect of elevated temperatures (room temperature and 750 °C) on the tensile properties was investigated. In this work, it was shown that alloy 21-6-9 could be built successfully using LPBF, with good properties and a density of 99.7%, having an ultimate tensile strength of 825 MPa, with an elongation of 41%, and without any significant anisotropic behavior.

## 1. Introduction

Additive manufacturing (AM) has over the past decade gained more and more traction as an alternative manufacturing route for certain metallic components (e.g., steel, titanium, and nickel alloys) [1,2]. Although the term AM encompasses numerous different processes, one of the more popular ones that have emerged is laser powder-bed fusion (LPBF) [3]. In LPBF, the build is realized by slicing a 3D model of the component to be built into multiple slices (with a layer thickness of approximately 20–60 µm). The laser scans the build plate, which is covered in a one-layer-thick metal powder. The input data from each slice provide the machine with coordinates for local fusion to be carried out and proceeds to build each layer incrementally on top of the previously deposited layer (while the build plate is lowered with steps equal to layer thickness before each redistribution of powder) [4]. A major factor in the quality of LPBF-built material is the process parameters used during manufacturing—they have been shown to heavily influence properties such as porosity, lack of fusion (LOF), grain size, and texture [5,6,7,8]. Due to the complexity of LPBF, where many process parameters influence the build quality, a formula has been created to estimate the energy density (J/mm^3^) introduced into the material during building [9]:

(1) $$E\;\left[\frac{J}{mm^{3}}\right]=\frac{P}{v*h*t}$$

Equation (1) accounts for laser power (*P* (W)), hatch distance (*h* (mm)), scan speed (*v* (ms^−1^)), and layer thickness (*t* (mm)), which, based on previous findings, have been shown to be an effective method to find outer boundaries for potential LPBF process parameters. Below a certain threshold, energy input may be insufficient to melt all the scanned powder during building, resulting in increased porosity, LOF, cracks, and balling. However, if the energy density is above a certain threshold, irregularities in the scan track where porosity and spherical balls (spatter from the overheated-liquid phase) can introduce defects and reduce part density [5,10]. Looking at individual process parameters, it has been reported that one of the most influential parameters for high relative density of a built LPBF component is laser power, while hatch distance did not cause significant changes in relative density (in the investigated range, 100 W ≤ P ≤ 150 W and 0.05 mm ≤ h ≤ 0.07 mm) [11]. However, other authors report that a hatch distance of 0.12 mm caused pore formation and decreased relative density of their LPBF-built material when comparing two builds with the same energy density (energy density was kept constant by compensating with an increase/decrease of scan speed) [12].

The investigated alloy, 21-6-9, is a nitrogen-strengthened austenitic stainless steel alloy, mainly comprised of (nominal amounts according to AMS 5656) 21 wt% Cr, 6 wt% Ni, 9 wt% Mn, and 0.15–0.4 wt% N. When comparing 21-6-9 to the 300 series of austenitic stainless steels, there are major compositional differences: the lower Ni content in 21-6-9 (the principal austenite stabilizer in the 300 series of austenitic stainless steels) is due to the reduced solubility of N in the alloy with increasing Ni content [13], and the austenite stability is therefore achieved by alloying with Mn and N, both of which are effective austenite-stabilizing elements (chromium equivalence of 0.5 and 30, respectively [14]). Solid-solution strengthening by nitrogen addition increases both yield and tensile strength, thus giving it an advantage in certain applications compared to many of the nitrogen-lean austenitic stainless-steel alloys [15], while still reaping the benefits of having a face-centered cubic (FCC) crystal structure that facilitates slip even at cryogenic temperatures [16]. Alloy 21-6-9 has found its main usage in aerospace and cryogenic industrial applications [17,18,19]; this is mainly due to a comparably high yield and tensile strength (relative to the nitrogen-lean austenitic stainless steels), thermal stability at high temperatures, and retained toughness down to cryogenic temperatures (relative to martensitic and ferritic stainless steels) [20,21].

Being able to manufacture 21-6-9 via LPBF would enable small-batch production of complex near-net-shaped components for the aerospace industry, which could decrease lead times and reduce weight, a critical factor for any component built to fly [22]. For some of the more common austenitic stainless-steel alloys (e.g., 304L, 316L), extensive studies have been carried out to investigate the effect of process parameters during LPBF processing [3], which cannot be said for 21-6-9. Previous research on LPBF manufacturing of various alloys has illuminated the importance of finding the individual optimal parameters for the material at hand, where large discrepancies can be caused by minor differences in process parameters, build environment, and powder quality [23,24,25]. The LPBF process is intrinsically complex; optimizing build quality is not only a matter of process parameters (e.g., compositional deviations, part geometry), but it is a vital foundation for realizing high-performance LPBF-built parts and thus an interesting initial research angle.

## 2. Experimental Method

### 2.1. Material Design of Experiment

The material investigated in this work was manufactured using an SLM Solutions 125 machine in an argon atmosphere. For the experimental setup of the process parameters for the 21-6-9 stainless steel, a design of experiment (DOE) approach was performed using the software MODDE. A full factorial design (2 levels) was applied and the hatch distance, layer thickness, laser power, and scan speed were altered, and the aim was to minimize the porosity fraction. As a reference point for the selection of intervals for the parameters, a parameter set for both layer thicknesses from SLM Solutions for 316L stainless steel was used and these parameter sets (samples 12 and 24) were also added to the experimental matrix. The process parameters that were used are shown in Table 1, where samples 12 and 24 are the parameters for 316L stainless steel. The experiment was divided into two print jobs due to the two different layer thicknesses. Therefore, the three center points from the DOE were applied for both layer thicknesses, see samples 9-11 and 21-23 in Table 1. From early experiments, failure during printing occurred and the edges of the printed parts were sticking up through the powder bed. Therefore, it was decided to run the DOE without any contouring parameters to be able to optimize the core. For this work, 24 samples were manufactured in two batches.

**Figure 1:**
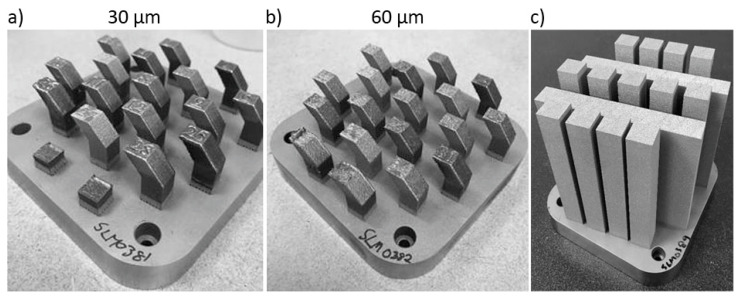
Overview of the samples attached to the build plate. Two batches were built, with 30 µm (see (**a**)) and 60 µm layer thickness respectively (see (**b**)). With the optimized process parameters from the design of experiment (DOE) the build seen in (**c**) was built.

In Figure 1, the DOE samples are shown in (a) and (b), where (a) corresponds to 30 µm layer thickness and (b) 60 µm layer thickness. The quadratic base of each sample was 10 × 10 mm. With the process parameters 180 W power, 900 mm/s scan speed, 30 µm layer thickness, and 0.14 mm hatch distance (process parameter set no. 7), the blanks and walls shown in (c) were built. The walls in (c) were used for making horizontal tensile specimens (the major axis is horizontal to the build plate), while the vertical tensile specimens are perpendicular to the build plate.

Each sample was mounted into a resin and ground and polished until mirror-like surfaces were obtained. Then the density (i.e., 100 minus porosity) was measured by first obtaining a large-area stitched image (100 mm^2^) per sample by using an AX10 (ZEISS, Oberkochen, Germany) light optical microscope (LOM). These images were then analyzed using the image software ImageJ, version 1.52a, [26] to obtain the area fraction of porosity, i.e., black pixels correspond to porosity and white fused 21-6-9. Once the area fraction of porosity had been measured, the max defect length was measured using the ImageJ software. Vickers hardness measurements were performed using a Duramin-40 (Struers, Cleveland, OH, USA) machine. The applied load was set to 1 kg with a dwell time of 15 s. The hardness measurements were performed on material that had been polished, and the indentations were positioned in a way that porosity would not spoil indent quality. The number of indents per sample was 7, and these indents were made randomly distributed in the bulk of the material.

### 2.2. Microstructure Characterization and Fractography

To reveal the microstructure of the material, electrolytic etching was performed (oxalic acid 10%, 3 V, 50 s), and then the grain sizes were measured following the ASTM standard E112-13, using the line-intercept method. Before the electron backscattered diffraction (EBSD) measurements, the material was polished using vibration polishing while being immersed in colloidal silica (0.04 µm) for 5 h. The scanning electron microscope (SEM) used was a Gemini 450 (ZEISS), equipped with an EBSD detector. The detector used was a Symmetry detector from Oxford Instruments. The acceleration voltage was set to 15 kV with a current of 1.2 nA. For the inverse-pole-figure (IPF) maps, a step size of 2 µm was utilized. Post-processing of the images was performed using Oxford Instruments software Aztec Crystal, where 5 neighboring grains were used to determine the unindexed pixels. This software was also utilized to obtain the preferred crystallographic direction in the material (i.e., multiples of random distribution (MRD) values). The IPF maps were also used for verifying the grain size measurements obtained using optical microscopy. All the fracture surfaces were investigated and the fractography was performed using an EVO 50 SEM (ZEISS).

### 2.3. Tensile-Strength Measurements

Prior to tensile testing, the specimens were heat-treated at 600 °C for 2 h and then cooled in the furnace. The main purpose of this heat treatment was to relieve potential residual stresses. The tensile testing was performed at Metcut Research Inc. in Cincinnati, OH, USA, and the blanks were machined according to the drawing MRI 1512, with gage dimensions 6.35 mm diameter times 31.75 mm length. The testing was performed according to standards ASTM E8 (16) and ASTM E21 (17) at room temperature (RT) and 750 °C. The temperature of 750 °C was chosen due to its interest industrially; for the elevated temperature testing, it took 15 min to go from 24 to 750 °C. The tests were carried out in strain control at a strain rate of 0.5%/min beyond 0.2% yielding followed by displacement control at a crosshead speed of 0.05mm/mm gauge length/min to failure.

## 3. Results

### 3.1. Density Measurements

**Figure 2:**
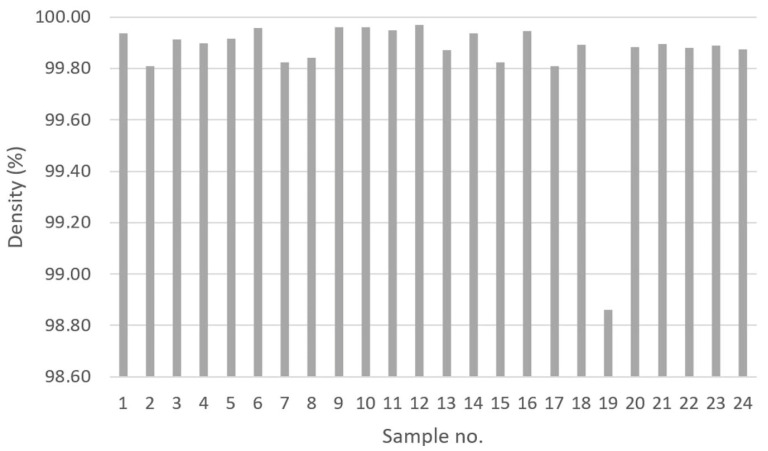
The density values for the investigated samples. The densest sample was no. 12, with a value of 99.97%. The most porous was no. 19 with a density value of 98.86%.

In Figure 2, the density of the investigated samples is shown. The four sets of process parameters that rendered the highest relative densities were samples no. 6, 9, 10, and 12 with no. 12 having 99.97% and the others 99.96%. The lowest density was found for sample no. 19 with a value of 98.86%. Samples 9 to 11 and 21 to 23 were built using identical process parameters (to verify repeatability), and no large variation in density was observed.

**Figure 3:**
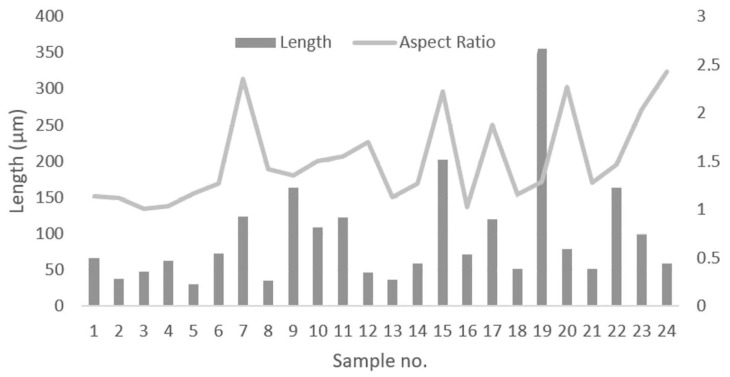
Max defect length in µm for the vertical cross-section along with the aspect ratio (length/width).

The maximum individual defect length is presented in Figure 3. The largest defect length was found for sample no. 19, being 355 µm. The smallest was found for sample no. 5 with 30 µm. Note that some samples, such as no. 9 and 10, that had high densities also had large defects as shown in Figure 3.

**Figure 4:**
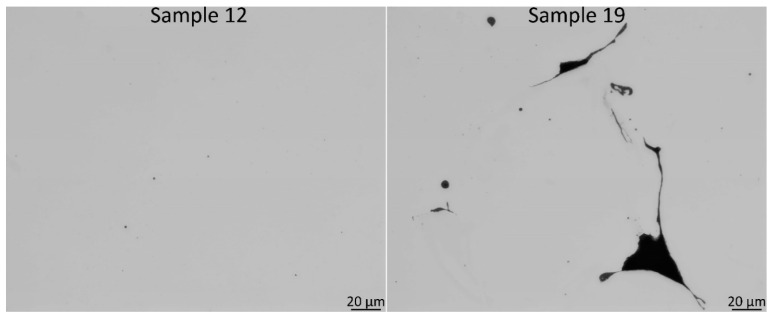
Overview of a polished cross-section of sample 12 (the densest material) and sample 19 (the most porous). For sample 12, the defects were spherical porosities, whereas for 19, it was both the spherical porosities and LOF.

In Figure 4, an overview of the material with the highest density (no. 12) and lowest (no. 19) are shown. For no. 12, there was mainly a presence of smaller spherical porosities, whereas for no. 19, both spherical porosities and larger LOF defects were present.

### 3.2. Microstructure Characterization and Hardness

**Figure 5:**
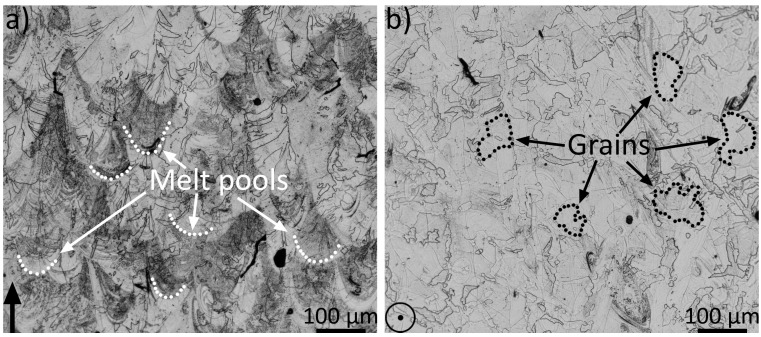
Two micrographs showing the vertical cross-section, (**a**), and horizontal, (**b**). The arrow in a) and the circle in (**b**) show the build direction. In (**a**), the melt pools are visible in the material, where the white dots exemplify some melt pools. Examples of grains are visualized using black dots in (**b**). Both micrographs originate from sample no. 7.

In Figure 5, the microstructure of sample no. 7 in the vertical (a) and in the horizontal (b) directions is shown. In (a), the melt pools were visible, indicated with white dotted lines. The grain sizes are exemplified in (b) with black dotted lines. Porosity (seen as black areas) was present in the material.

**Figure 6:**
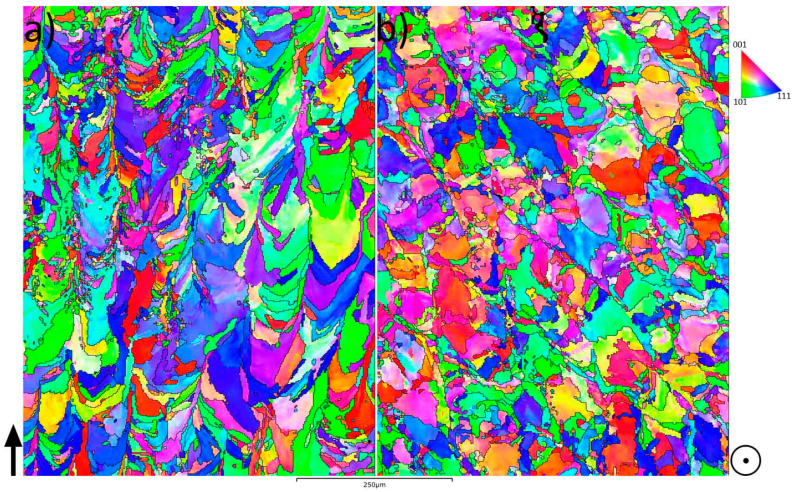
The inverse-pole-figure maps of the vertical, (**a**), and the horizontal, (**b**), cross-sections. The black arrow in (**a**) shows the build direction, whereas in (**b**), the build direction is in the plane of the paper. Both IPF maps originate from sample no. 7.

Figure 6 shows the IPF maps for the two investigated directions, vertical in (a) and horizontal in (b), i.e., perpendicular and parallel to the AM layers, respectively, for sample no. 7. From the EBSD measurement, the texture was obtained and the multiples of random distribution (MRD) for the horizontal cross-section was 3.64, while for the vertical cross-section, it was 3.97.

**Figure 7:**
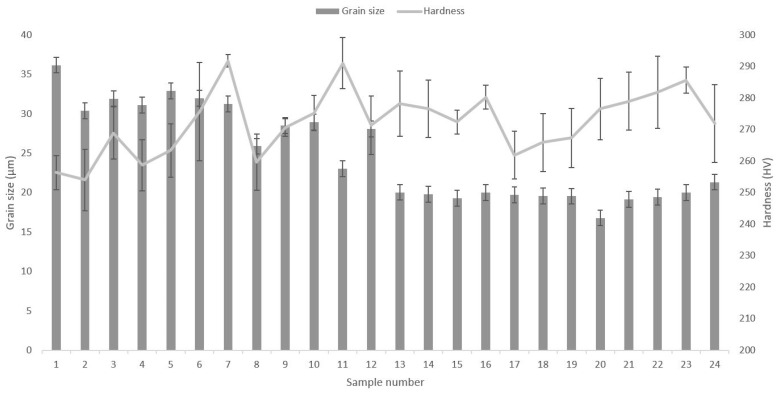
Grain size (see columns) and hardness (see line) for the 24 investigated samples. The coarsest grain size was found for sample 1, whereas the finest grain size for sample no. 20. The highest hardness was found for sample no. 11 (roughly equal to that of sample 7).

Figure 7 shows the results of the grain size measurements and the hardness. The highest hardness was found for sample no. 7, having a hardness value of 292 ± 2 HV. The lowest hardness was found for sample 2, having a hardness of 215 ± 10 HV. The material with the finest grain size was found for build parameters no. 20, with a mean intercept distance of 17 µm. The coarsest material was found for sample 1, having a grain mean intercept distance of 36 µm.

### 3.3. Tensile Properties

**Figure 8:**
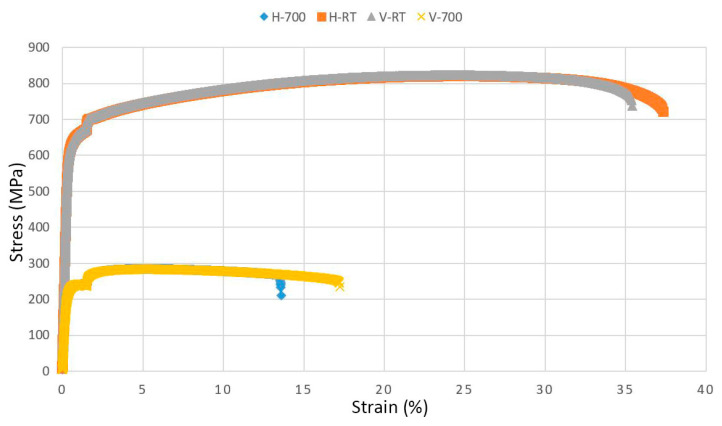
Stress–strain curve for the horizontal (H) and vertical (V) material at two temperatures, 750 °C and RT. No anisotropic behavior was observed between the two directions.

As can be observed in Figure 8, the vertical sample tested at RT had an ultimate tensile strength (UTS) of 825 MPa and an Rp_0.2_ of 620 MPa. The measured Young’s modulus was 192 GPa, and the elongation was 41%. At 750 °C, the UTS was 284 MPa and Rp_0.2_ 225 MPa. The measured Young’s modulus was 102 GPa and the elongation 21%. No significant anisotropic behavior was observed. For the horizontal specimens, the properties were similar at RT, being 820 MPa in UTS, 620 MPa YS, 44% elongation, and a Young’s modulus of 188 GPa. For the testing at an elevated temperature, no anisotropic behavior could be observed for these conditions either, being 284 MPa in UTS, 226 MPa in YS, 16% elongation, and 116 GPa in Young’s modulus.

### 3.4. Fractography

**Figure 9:**
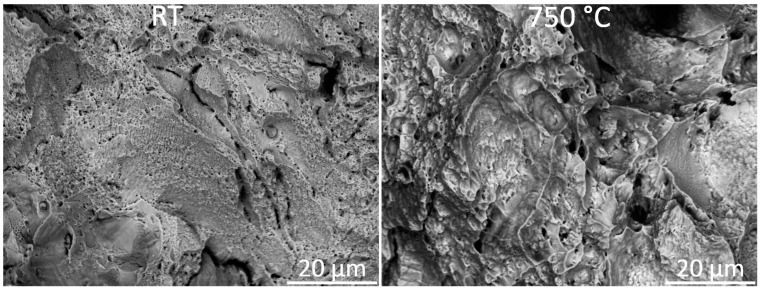
Representative fracture surfaces of the vertical samples tested at RT and 750 °C. At RT, the dimples were smaller compared to the material tested at 750 °C.

The fractography revealed a difference in dimple size between the tensile specimens tested at RT and 750 °C. At RT, the dimple size was small, whereas at the elevated temperature, the dimples were larger. This dimple size difference between the two temperatures is shown in Figure 9.

## 4. Discussion

Alloy 21-6-9 is a stainless steel with a microstructure constituting austenitic grains. With LPBF, the heat is applied sequentially, with a heat flow toward the building plate, i.e., normal to the build direction [27]. This produces a steep thermal gradient in the sample. As a result, the grains at the sample’s edge begin to grow upward and large elongated grains form in the middle, with an epitaxial growth [11]. The solidification mainly depends on two parameters, i.e., thermal gradient G and the solidification growth rate R. G/R ratio defines the microstructure morphology, and G*R defines the cooling rate and hence the refinement of the microstructure; as the G*R value increases, the grain size becomes finer [5,28]. As shown in the IPF maps in Figure 6, the typical morphology of the columnar microstructure of AM [29] is observed in the vertical cross-section, whereas a more equiaxed morphology is observed in the horizontal. All four investigated process parameters have a cumulative effect on the energy. Energy density determines the cooling rate, and there is a correlation with grain size, where samples 1 to 12 (generally higher energy densities) produced coarser grains than samples 13–24 (lower energy densities). The reason for the difference in grain size is due to thermal input having a direct effect on the cooling rate of the fused material, where higher energy densities reduce the cooling rate which in turn allows for additional grain coarsening [5,27]. No direct relationship between the grain sizes and hardness values could be observed. As shown in Figure 7, the hardness was somewhat constant for the various samples, given their standard deviations.

There are more than a dozen parameters that affect the quality of the parts produced using AM. To reach full density, it is required to balance laser power, scan speed, beam size, layer thickness, and other parameters [30]. In this work, laser power, scan speed, hatch distance, and layer thickness were the varying parameters for influencing the sample’s density. Scan speed has a major influence on productivity and densification. In this experiment, it was observed that for the same laser power (180 W), hatch distance (0.1 mm), and layer thickness (30 µm), as the scan speed increased, the density of the samples reduced as there was not enough time for complete fusion. Out of the three scan speeds investigated, the samples with 800 mm/s showed improved density compared to the others. If the scan speed is increased, the density decreases due to reduced fusion, while if the speed is decreased too much, gas porosity can form due to vaporization [30]. Another observation was that as the hatch distance increased, the density was reduced since an increase in the hatch distance decreases the overlap of melt pools. This correlates well with Thijs et al. [31], who observed that as the overlap between the melt pool decreases, the defect size increases, i.e., reduction in density. Increasing the layer thickness can increase the productivity, but it may reduce the density of the part as well.

One concern prior to building 21-6-9 with LPBF was how the nitrogen content in the steel would be affected because 21-6-9 is a nitrogen-strengthened stainless steel and it could be possible for nitrogen to be dispersed from the material during the melting. If nitrogen is dispersed, the strength will be reduced as nitrogen acts as an interstitial strengthener. However, the LPBF-built 21-6-9 showed higher YS and UTS (620 and 825 MPa, respectively) compared to wrought 21-6-9 (336 and 706 MPa) [32]. Thus, the concern of nitrogen depletion was not legitimate. The reason for increased strength compared to the wrought material is the refinement of grain size, Torres [32] reported an average grain size of 60 µm, while the grain size for the LPBF-processed material investigated herein was half that size. The ductility of the LPBF-built 21-6-9 was shown to be lower than the wrought counterpart. Bajaj et al. [33] concluded that for AM-built steels to obtain the same ductility as conventionally manufactured steels, fracture initiation sites such as microcracks and LOF must be eliminated as they have been shown to influence the ductility negatively.

As expected, all specimens tested at RT showed higher ultimate tensile strength, yield strength, and elongation compared to the specimens tested at 750 °C, as reported by several authors for both 316 and 21-6-9 stainless steel [32,34,35]. S. Dryepondt et al. [34] attributed these results to the presence of twins. At RT, twins were observed in LPBF-built 316L, whilst at higher temperatures, these twins were lacking. Twinning causes the formation of twin boundaries due to changes in plane orientation that facilitates slip to occur. These twin boundaries are important as they have lower energy than high-angle boundaries, allowing them to store dislocations more efficiently, enhancing strength and ductility [27,36]. Another explanation for the reduced strength and ductility at elevated temperatures was given by Zhong et al. [37]. Deformation is hindered by the pinning effect of dislocations and nano-inclusions, which results in the strengthening of the material properties at low temperatures. However, when the temperature increases, the energy needed for dislocation motion decreases which in turn weakens the pinning effect, resulting in reduced tensile properties.

The fracture surface morphologies of LPBF 21-6-9 at RT and 750 °C are shown in Figure 9. A difference in dimple size can be observed for the two test temperatures: at RT, there were many smaller-sized dimples whereas at elevated temperatures, fewer and larger dimples were present. The same relationship between dimple size and tensile properties at RT and elevated temperatures has been reported by Choudhary and Kondisetti et.al [38,39]. Zhong et al. [37] attributed this variation in dimple size to that debonding of material experiences more resistance at lower temperatures than at elevated temperatures, again explained by the pinning effect of dislocations and nano-inclusions.

When choosing the process parameter set to use, several parameters need to be taken into consideration, such as build rate, microstructure, density, surface roughness, etc. From the results of the study, process parameter set no. 20 rendered good material, combining the highest build rate with the finest grain size while maintaining a density of 99.84%. In this work, the tensile specimens were built with process parameter set no. 7 before the complete characterization was finished. Still, this process parameter set showed the highest hardness, best build rate out of the 30 µm layer process parameters, and a density of 99.82%. Fatigue strength, though, is more dependent on the number of defects than tensile strength; thus process parameter set no. 12 is likely preferable for that application with its density of 99.97%, given that hot isostatic pressing is not available.

## 5. Conclusions

In this work, the effect of process parameters for laser powder-bed fusion (LPBF)-built 21-6-9 stainless steel on density, grain size, and hardness was investigated. Twenty different sets of process parameters were investigated. The tensile properties were investigated for one set of process parameters, investigating the effect of varying temperature (room temperature and 750 °C) and build orientations (vertical and horizontal).

- The material with the best density was obtained using process parameter set no. 12, i.e., 200 W power, 800 mm/s scan speed, 30 µm layer thickness, and 0.12 mm hatch distance. The density for this process parameter set was 99.97%.
- The smallest max defect length was observed for process parameter set no. 5 (see Table 1).
- The finest grain size was obtained using process parameter set no. 20 (highest build rate out of 30 and 60 µm layer thickness).
- The highest hardness was obtained using process parameter set no. 7 (highest build rate out of 30 µm layer thickness).
- The ultimate tensile strength of LPBF-built 21-6-9 was 825 MPa, with an elongation at failure of 41%. At a temperature of 750 °C, the tensile strength was 284 MPa in UTS and 225 MPa, with an elongation at failure of 16%.
- No anisotropic behavior was observed between the vertical and horizontal directions.

## Figures and Tables

**Table 1:** For samples 1 to 12, a layer thickness of 30 µm was utilized and for 13-24 60 µm. The hatch distance, laser power, and velocity were altered, varying each process parameter with three settings, which rendered variations in line energy and energy density. The build rate is scan speed times layer thickness (in mm) times hatch distance.

Sample no.	Laser Power (W)	Scan Speed (mm/s)	Layer Thickness (μm)	Hatch Distance (mm)	Line Energy (J/mm)	Energy Density (J/mm^3^)	Build Rate (mm^3^/s)
1	180	700	30	0.10	0.257	85.71	2.1
2	300	700	30	0.10	0.429	142.86	2.1
3	180	900	30	0.10	0.200	66.67	2.7
4	300	900	30	0.10	0.333	111.11	2.7
5	180	700	30	0.14	0.257	61.22	2.9
6	300	700	30	0.14	0.429	102.04	2.9
7	180	900	30	0.14	0.200	47.62	3.8
8	300	900	30	0.14	0.333	79.37	3.8
9	240	800	30	0.12	0.300	83.33	2.9
10	240	800	30	0.12	0.300	83.33	2.9
11	240	800	30	0.12	0.300	83.33	2.9
12	200	800	30	0.12	0.250	69.44	2.9
13	180	700	60	0.10	0.257	42.86	4.2
14	300	700	60	0.10	0.429	71.43	4.2
15	180	900	60	0.10	0.200	33.33	5.4
16	300	900	60	0.10	0.333	55.56	5.4
17	180	700	60	0.14	0.257	30.61	5.9
18	300	700	60	0.14	0.429	51.02	5.9
19	180	900	60	0.14	0.200	23.81	7.6
20	300	900	60	0.14	0.333	39.68	7.6
21	240	800	60	0.12	0.300	41.67	5.8
22	240	800	60	0.12	0.300	41.67	5.8
23	240	800	60	0.12	0.300	41.67	5.8
24	275	700	60	0.12	0.393	54.56	5

## Data Availability

Data sharing not available.
